# *In Vivo* Screening of Hepatocellular Carcinoma Using AC Susceptibility of Anti-Alpha Fetoprotein-Activated Magnetic Nanoparticles

**DOI:** 10.1371/journal.pone.0046756

**Published:** 2012-10-08

**Authors:** Jen-Jie Chieh, Kai-Wen Huang, Yang-De Lee, Herng-Er Horng, Hong-Chang Yang, Chin-Yih Hong

**Affiliations:** 1 Institute of Electro-optical Science and Technology, National Taiwan Normal University, Taipei, Taiwan; 2 Department of Surgery and Angiogenesis Center, National Taiwan University Hospital, and College of Medicine, National Taiwan University, Taipei, Taiwan; 3 Department of Electro-Optical Engineering, Kun Shan University, Tainan, Taiwan; 4 Graduate Institute of Biomedical Engineering, National Chung Hsing University, Taichung, Taiwan; Mount Sinai School of Medicine, United States of America

## Abstract

With antibody-mediated magnetic nanoparticles (MNPs) applied in cancer examinations, patients must pay at least twice for MNP reagents in immunomagnetic reduction (IMR) of *in vitro* screening and magnetic resonance imaging (MRI) of *in vivo* tests. This is because the high maintenance costs and complex analysis of MRI have limited the possibility of *in vivo* screening. Therefore, this study proposes novel methods for *in vivo* screening of tumors by examining the AC susceptibility of bound MNPs using scanning superconducting-quantum-interference-device (SQUID) biosusceptometry (SSB), thereby demonstrating high portability and improved economy. The favorable agreement between *in vivo* tests using SSB and MRI demonstrated the feasibility of *in vivo* screening using SSB for hepatocellular carcinoma (HCC) targeted by anti-alpha fetoprotein (AFP)-mediated MNPs. The magnetic labeling was also proved by *in vitro* tests using SSB and biopsy assays. Therefore, patients receiving bioprobe-mediated MNPs only once can undergo *in vivo* screening using SSB in the future.

## Introduction

Magnetic nanoparticles (MNPs) with bioprobes have recently been applied for screening by immunomagnetic reduction (IMR) [1], image contrast of magnetic resonance imaging (MRI) [2]–[3], hyperthermia [4]–[5], drug delivery [6]–[7], and surgical treatment [8] of tumors. Among these processes for examining tumors, only screening is employed for *in vitro* testing, whereas the other processes are employed for *in vivo* tests. Screening is limited to *in vitro* tests because the high cost and complex analysis of MRI discourages widespread use in clinics. Thus, diagnosed patients always pay at least twice for MNP reagents; the first payment is for the screening, involving more economical and facile IMR operation, and the second is for high-resolution MRI.

To compensate for the disadvantages of using MRI, multimodal MNPs [9]–[10] comprising MNPs with fluorochromes, radioactivity indicators, and bioprobes have been developed to increase the detection ability of MNPs using more economical and nonmagnetic methods than MRIs. However, the complex configuration of multimodal MNPs and other examination methods also increase costs and biological safety risks.

**Figure 1:**
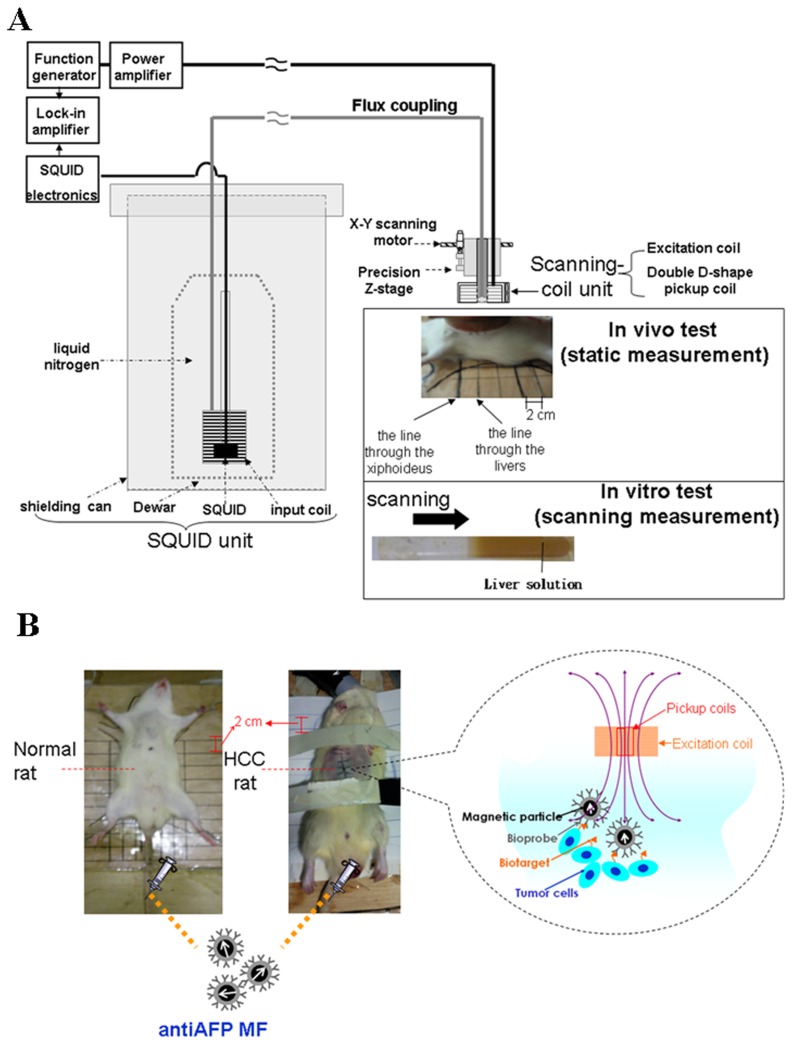
*In vivo* screening of HCC using SSB. (A) Experimental setup for the *in vivo* and *in vitro* tests; and (**B**) examination mechanism. Rats were arranged on the coordinates at 2 cm intervals. MF represents the magnetic fluid, and the arrows the magnetic particles, whose single magnetic moment varies with time.

The superior magnetic characteristics of MNPs used in *in vivo* examinations should not be limited to the principles of MNP-induced distortion of the MRI field. For example, the nonlinear response of magnetic particles was used for the novel method of magnetic particle imaging (MPI) [11]; however, because of its high-field properties and field configuration similar to that of MRI systems, the benefits of MNPs for *in vivo* examinations are also limited. Nevertheless, SSB (**Fig. 1A**) based on assessing the in-phase component of AC susceptibility has been approved for *in vivo* tracking of MNPs without antibodies [12]–[13]. Furthermore, because the heat of MNP hyperthermia is generated by the out-of-phase component of the AC susceptibility of MNPs [4], few MNPs bound with bioprobes on tumor tissue might be detected because of their weaker in-phase component of AC susceptibility (**Fig. 1B**).

This study examined the feasibility of using SSB to conduct *in vivo* screening of HCCs labeled with anti-AFP MNPs. The MRI results were also compared with those of SSB *in vivo* tests. Furthermore, the *in vitro* result obtained using SSB and biopsy tests were employed to verify the *in vivo* results. Additionally, SSB is an attractive option for *in vivo* screening because of its high portability and cost effectiveness.

## Materials and Methods

The Animal Care and Use committee of the College of Medicine, National Taiwan University, approved all experimental protocols (No. 20110009). All experiments were conducted according to the animal care guidelines of the university.

The anti-AFP magnetic fluid (MF) was synthesized by the covalent conjugation of anti-AFP antibodies on MNPs [14]. In this study, MNPs were composed of an Fe_3_O_4_ core and a dextran coating (MagQu Corp, New Taipei, ROC) [15]. The feasibility of using anti-AFP MFs for assaying AFPs in the plasma was verified using IMR with similar clinical applications [16]. In this study, anti-AFP MF was injected into 2 types of rats (normal rats and HCC rats). The HCC rats were male F344/NNarl rats (obtained from the National Laboratory Animal Center, Taipei, Taiwan, ROC) injected with the GP7TB cell line into their livers after three weeks. GP7TB is a rat liver epithelial tumor cell line with characteristics of liver stem-like cells that can develop into a tumor in F344/NNarl rats [17]. The anti-AFP MF dose for 2 normal rats (Rat A and Rat B) and 3 HCC rats (Rat C, Rat D, and Rat E) was 0.3 emu/g in 0.9 ml, equivalent to 30 mg/kg of iron according to a scale presented in other studies [18]–[19].

**Figure 2:**
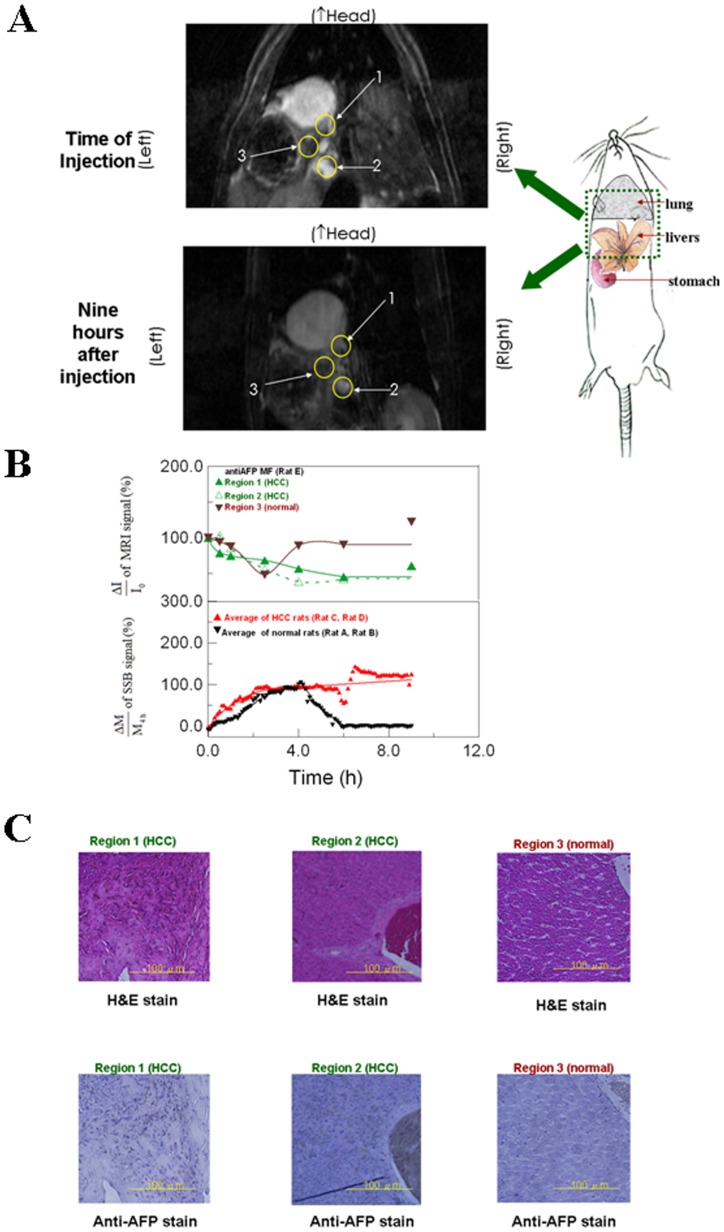
*In vivo* examination of anti-AFP MNPs in the livers between by MRI and by SSB. (A) MRI images at the time of injection and nine hours after injection in Regions 1 and Region 2, (representing HCC tissue) and Region 3 (representing normal tissue with yellow outlines). (B) Analysis of ΔI/I_0_ using MRI in Regions 1, 2, and 3 for Rat E and of ΔM/M_4h_ using SSB for Rats A and B, and Rats C and D. Spline smoothing with a spline tension of 2 was used to fit the data of one region and the data average of two rats for ΔI/I_0_ and ΔM/M_4h_, separately. (C) Optical images of the H&E staining and anti-AFP staining in Regions 1, 2, and 3 for Rat E at nine hours after injection. MF represents the magnetic fluid.

SSB (**Fig. 1A**) includes the SQUID sensor unit (JSQ Magnetometer, Julich, Germany), scanning coil unit composed of excitation and double D-shaped pickup coils, and copper wire for flux coupling. For the applied AC field, the product of the 400 Hz excitation frequency and 120 Oe field strength was approximately 3.82×10^3^ kA/m·s, and is smaller than the biological safety criterion of 4.85×10^8^ kA/m·s [20]. Additionally, the flux coupling was transferred from the pickup coils to the SQUID sensor, protected inside the shielding can and cooled using liquid nitrogen.

The xiphoideus of the rats, which were anesthetized using an inhalation system, was aligned with one of the coordinate lines on the wooden plate. Thus, the SSB coil unit was scanned across the torso along the coordinate line through the center of the livers (**Fig. 1A**), with an interval of 2 cm from the xiphoideus to determine the position of maximal magnetic intensity. Consequently, the SSB coil unit statically and continuously measured the magnetic intensity at the position of maximal magnetic intensity before and after the anti-AFP MFs injection [12]–[13], with zero time being the time of injection. The measured in-phase sample intensity is proportional to the magnetization (expressed as M). However, the normalized magnetization variation ΔM/M_4h_, defined as the difference of the ΔM magnetization between any time and the time of injection normalized by the magnetization four hours after injection M_4h_, was used to compare injected normal rats and HCC rats.

**Figure 3:**
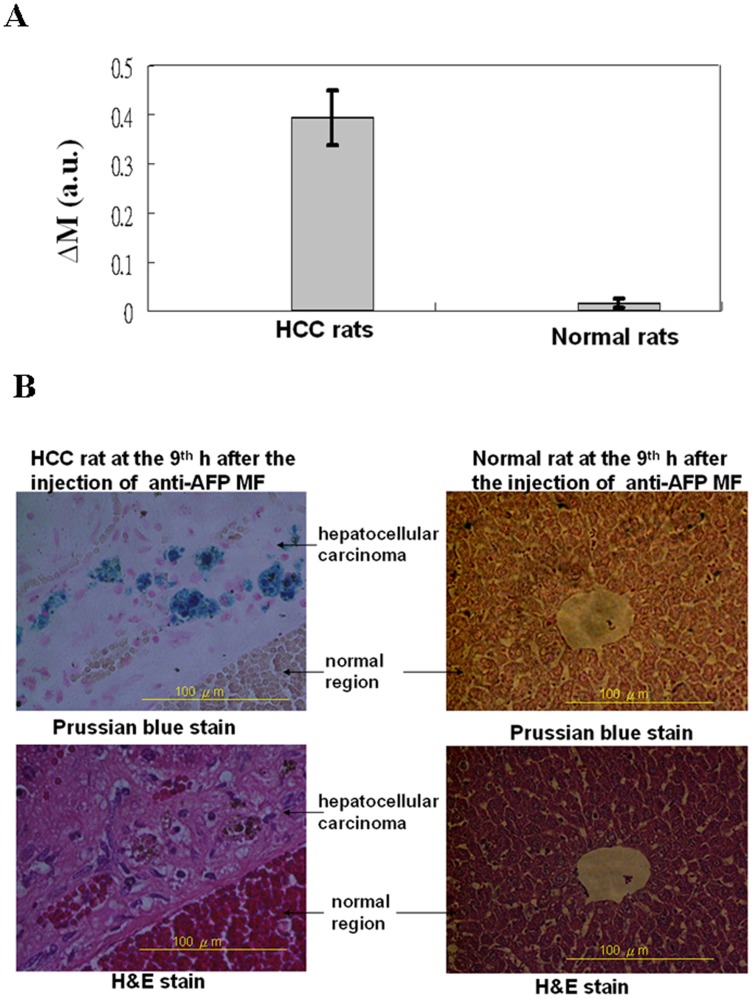
*In vitro* and biopsy tests. (A) Analysis of the ΔM of SSB for the differences between the magnetization nine hours after the time of injection; and (B) optical images of the H&E staining and Prussian blue staining for the liver tissue nine hours after the time of injection. MF represents magnetic fluid.

For MRI examination, a 3-T MRI system (Bruker, Ettlingen, Baden-Württemberg, Germany) was used for T_2_-weighted axial images at 1 mm intervals. Rat E was examined before and after receiving an anti-AFP MF injection. Three liver regions (Region 1, Region 2, and Region 3, **Fig. 2A**) were selected and marked with a yellow circle to compare the average intensity (expressed as I) of the entire circle before and after the anti-AFP MF injection. Background black was used as the reference signal for MRI intensity. The normalized intensity variation ΔI/I_0_ (defined as the ΔI intensity difference between any time and the time of injection over the initial intensity at the time of injection I_0_) was used for Regions 1 and 2 (representing the HCC tissue), and Region 3 (representing normal tissue) for comparison using SSB *in vivo* testing.

The livers of the examined rats euthanized at the ninth hour were processed for both the biopsy test and *in vitro* tests employing SSB. A biopsy test was conducted (Laboratory Animal Center, National Taiwan University, Taipei, Taiwan, ROC) for H&E staining, Prussian blue staining, and anti-AFP staining. The H&E staining was used to identify hepatic tissue or normal tissue, whereas the Prussian blue and anti-AFP staining were used to verify the magnetic labeling of *in vivo* tests. Optical images (at 400x magnification) were examined using a light microscope.

In the SSB examination, liver tissue was homogenized with water at a ratio of 0.3 to 0.4 g and poured into a glass tube [12]. After scanning the sample using SSB, the scanning wave area was used to analyze the anti-AFP MNP concentration, denoted as ΔM as a function of time (in hours) after injection. In this study, the liver tissues of normal and HCC rats at the time of injection were obtained from euthanizing another two of both types of rats without injecting anti-AFP MFs.

## Results

The *in vivo* results obtained using SSB (**Fig. 2B**) show that the normalized magnetization ΔM/M_4h_ for the livers of normal and HCC rats increased rapidly within the first two hours following anti-AFP MF injections and remained at the maximal level until the fourth hour. However, for normal rats, ΔM/M_4h_ decreased to its initial value after approximately the sixth hour, and this value was subsequently maintained. For HCC rats, ΔM/M_4h_ continued to increase gradually after the fourth hour.

The MRI results for Rat E following anti-AFP MF injection are shown in **Fig. 2B**. ΔI/I_0_ for the HCC tissue in Regions 1 and 2 decreased to the lowest level at approximately the fourth hour and remained at this level. However, ΔI/I_0_ in the normal tissue in Region 3 returned to its initial value at the fourth hour. Similarly, the binding of anti-AFP MNPs to HCC tissue and the metabolism of anti-AFP MNPs in normal tissue explain the phenomenon in Regions 1 and 2, as well as Region 3 separately. **Fig. 2C** shows the HE and anti-AFP staining of Regions 1, 2, and 3 in the MRI images at the tenth hour. They show that anti-AFP MNPs, (represented by brown spots in the macroscopic anti-AFP staining photos) accumulated in HCC sites for the area ratio, defined as the area of brown spots over of the whole macroscopic photo (approximately 5–10%). This proves the superior sensitivity of SSB for the detection of few anti-AFP MNPs in livers (around several 10^−2^ emu/g, which was obtained by calibrating the results of SSB *in vitro* test with the SSB results of anti-AFP MFs possessing known M).

To verify the *in vivo* results, the liver tissues from both HCC and normal rats were examined using *in vitro* SSB tests and biopsy tests at the ninth hour. For *in vitro* SSB testing (**Fig. 3A**), ΔM shows that the MNPs were distributed in large numbers in the HCC tissue, although almost none were observed in normal tissue. Furthermore, only MNPs that were targeted on HCC sites could express AC susceptibility, although biodegraded iron ions stored in normal livers could not [13]. **Figure 3B** shows the images of both H&E and Prussian blue staining for the liver tissue from both normal and HCC rats nine hours after anti-AFP MF injections. In the H&E staining, cells with clear and complete architectures were observed in the liver tissue of the normal rats, and these cells also developed in proximity to disorganized architectures in the HCC rats because of the increased ratio of nuclei to cytoplasm. The Prussian blue staining of the same tissues showed numerous blue spots that represented anti-AFP MNPs in the HCC tissue. These blue spots did not appear in the normal tissue.

## Discussion

**Fig. 2B** shows that the time available to metabolize anti-AFP MFs is six hours. The ΔM/M_4h_ differences between the normal and HCC rats began after four hours and lasted until at least the fifth hour. Furthermore, the MRI results showed opposing negative variations because the examination principles were different from those used in SSB. Additionally, the metabolism time of four hours in MRI analysis is shorter than the six hours in SSB examination. A shorter metabolism time in MRI may result from the image analysis of a smaller region of approximately 5 mm in diameter, in contrast to the intensity analysis of the SSB measurement region with the diameter of several centimeters, which is adequately large to represent the entire livers.

For the magnetic intensity variation of the HCC tissue, ΔM/M_4h_ using SSB increased after the fourth hour because of an increase in the accumulation of anti-AFP MNPs in the livers. However, constant ΔI/I_0_ in Regions 1 and Region 2 using MRI was maintained because of the possible binding saturation of anti-AFP MNPs in small and local regions. The results show that after the fourth hour, the difference of ΔM/M_4h_ using SSB increased, whereas the difference of ΔI/I_0_ using MRI remained constant. This finding indicates that *in vivo* screening of HCC tissue using SSB provides the superior characteristic of increasing the magnetic-labeling effect after the fourth hour, as compared to using MRI.

The favorable agreement between the *in vivo* and *in vitro* results demonstrates the feasibility of using SSB for *in vitro* and *in vivo* examinations of HCC labeled with anti-AFP MNPs. Additionally, the biopsy results are consistent with those of the *in vitro* test. The feasibility of *in vivo* screening of HCC using SSB was confirmed by conducting the gold standard biopsy test.

In conclusion, this study demonstrates that using SSB is suitable for *in vivo* screening and *in vitro* examinations. Compared to using MRI, *in vivo* screening of HCC labeled with anti-AFP MNPs using SSB is more cost-efficient, easier to conduct, and more significant. These advantages increase the popularity of *in vivo* screening and reduce the costs of MNP reagents for patients. The number of MNPs measured in tissues was consistent with that of the biopsy test. These results further demonstrate the feasibility of *in vivo* screening of HCC in animals.
